# Host-response biomarkers for precision tuberculosis: from immune transcriptomic signatures to clinical risk stratification

**DOI:** 10.3389/fcell.2026.1916291

**Published:** 2026-08-24

**Authors:** Nan Yan, Zhen Wang, Shilei Dong, Si Wang, Qinlong Sun, Jiusong Luan

**Affiliations:** 1 Department of Tuberculosis, Affiliated Hospital of Hebei University, Baoding, Hebei, China; 2 Department of Integrated Traditional Chinese and Western Medicine, Affiliated Hospital of Hebei University, Baoding, Hebei, China; 3 Department of Radiation Oncology, Affiliated Hospital of Hebei University, Baoding, Hebei, China; 4 Department of Infectious Diseases, Affiliated Hospital of Hebei University, Baoding, Hebei, China; 5 Department of Critical Care Medicine, Affiliated Hospital of Hebei University, Baoding, Hebei, China; 6 Department of Respiratory Medicine, Affiliated Hospital of Hebei University, Baoding, Hebei, China

**Keywords:** host-response biomarkers, immune signatures, precision medicine, recurrence, risk stratification, transcriptomic signatures, treatment monitoring, tuberculosis

## Abstract

Tuberculosis (TB) remains a major infectious cause of morbidity and mortality, while current diagnostic and treatment-monitoring pathways remain largely pathogen-centered. Microbiological tests are indispensable for confirming TB and detecting drug resistance, but they do not directly measure host inflammatory activity, disease-stage heterogeneity, risk of progression, biological response to therapy, or recurrence after treatment completion. Host-response biomarkers, particularly blood transcriptomic signatures, provide a complementary route toward precision TB medicine. Recent multicenter validation studies, household-contact cohorts, treatment-monitoring analyses, cell-free RNA profiling, single-cell studies, and recurrence cohorts have refined the translational evidence base for immune transcriptomic biomarkers. Across studies, active and progressive TB is characterized by interferon-inducible transcription, inflammatory myeloid remodeling, neutrophil and monocyte activation, antigen-specific T-cell activation, and partial normalization during effective therapy. These signals can support clinically relevant use cases, including sputum-independent triage of symptomatic patients, short-term prediction of progression among exposed contacts, identification of subclinical or incipient disease biology, treatment-response monitoring, and post-treatment recurrence detection. However, implementation is constrained by imperfect specificity against non-TB inflammation, heterogeneity across HIV, diabetes, pediatric, extrapulmonary and geographically diverse populations, assay standardization requirements, and limited interventional evidence. This Mini Review synthesizes recent evidence and proposes a focused translational framework linking immune transcriptomic mechanisms to actionable clinical risk stratification in TB.

## 1 Introduction

Tuberculosis (TB), caused by *Mycobacterium tuberculosis* (Mtb), remains a major global health challenge. The 2025 WHO global TB report provides the latest consolidated assessment of the global epidemic and emphasizes persistent gaps in prevention, diagnosis and treatment (World Health Organization, 2025). Current TB care is still dominated by pathogen-centered tools: sputum microscopy, culture, nucleic-acid amplification tests, molecular drug-resistance assays and drug-susceptibility testing. These tools are essential, but they cannot directly quantify immune activation, tissue-damaging inflammation, risk of progression from infection to disease, response biology during treatment, or the probability of recurrence after treatment completion (Nogueira et al., 2022). Host-response biomarkers are therefore intended to complement rather than replace pathogen detection by adding information on disease activity and clinically relevant risk stratification.

The biological problem is that TB is not a binary state of latent infection versus active disease. Instead, it spans Mtb exposure, immune sensitization, incipient TB, subclinical disease, symptomatic pulmonary disease, extrapulmonary disease, treatment response, residual pathology, relapse and reinfection. Blood transcriptomic signatures have become the most developed host-response biomarker class for interrogating this continuum. Systematic reviews and pooled analyses have shown that host blood RNA signatures can distinguish TB disease from other states and can predict short-term progression, although performance depends on population, time horizon and clinical context (Gupta et al., 2020; Mulenga et al., 2020). This Mini Review focuses on immune transcriptomic signatures as host-response biomarkers for precision TB and organizes the evidence around clinically actionable risk stratification. Transitions among these states are dynamic, heterogeneous and not necessarily linear or unidirectional. Unlike broader host-biomarker reviews, the present review is organized around specific clinical decisions and explicitly links immune mechanisms, comparator design, evidence maturity and implementation constraints.

### 1.1 Literature search strategy

For this targeted narrative Mini Review, PubMed/MEDLINE, Web of Science and Scopus were searched for English-language publications from January 2019 through June 2026, supplemented by landmark earlier studies. The principal Boolean structure combined (“tuberculosis” OR “*Mycobacterium tuberculosis*”) with (“host-response biomarker” OR “blood transcriptomic signature” OR “RNA signature” OR “cell-free RNA”) and (“diagnosis” OR “progression” OR “treatment monitoring” OR “recurrence”). Priority was given to peer-reviewed human cohorts, diagnostic-accuracy studies, longitudinal studies, trials, systematic reviews and meta-analyses; selected single-cell or experimental studies were included only to clarify biological mechanisms. Duplicate reports, non-TB studies and studies without a relevant host-response or clinical endpoint were excluded. This was a focused narrative synthesis rather than a formal systematic review. Throughout this review, ‘host-response transcriptomic signatures’ is used as the umbrella term, while ‘blood RNA’ and ‘cfRNA’ are retained only when specifying specimen or assay type.

## 2 Biological basis of host-response transcriptomic signatures

The rationale for host-response biomarkers is grounded in TB immunopathogenesis. Longitudinal blood transcriptomics show that active TB is associated with interferon-inducible genes, inflammatory myeloid activation and neutrophil-related modules, with gradual attenuation during successful treatment (Tabone et al., 2021). Parsimonious signatures such as RISK6 further support the concept that a small set of host transcripts can capture disease risk, diagnostic state and treatment-response dynamics across populations (Penn-Nicholson et al., 2020; Bayaa et al., 2021). These signatures do not measure bacillary burden directly; rather, they function as systemic proxies for the host-pathogen interaction.

Recent cell-resolved studies have clarified the cellular sources of these bulk blood signals. Prospective real-time PCR validation confirmed that published host blood signatures can be measured using clinically adaptable platforms (Mendelsohn et al., 2022), while sputum-independent RNA and protein studies showed that whole-blood transcriptional signals can complement non-sputum diagnostic strategies (Sivakumaran et al., 2021). Active TB signatures are strongly influenced by monocyte and neutrophil programs. Single-cell profiling indicates that distinct CD14^+^ monocyte subsets contribute substantially to active TB blood signatures, and neutrophil transcriptomic studies have identified disease-associated inflammatory programs that help explain the dominance of interferon-myeloid axes in bulk blood (Hillman et al., 2023; Geng et al., 2022).

**FIGURE 1:**
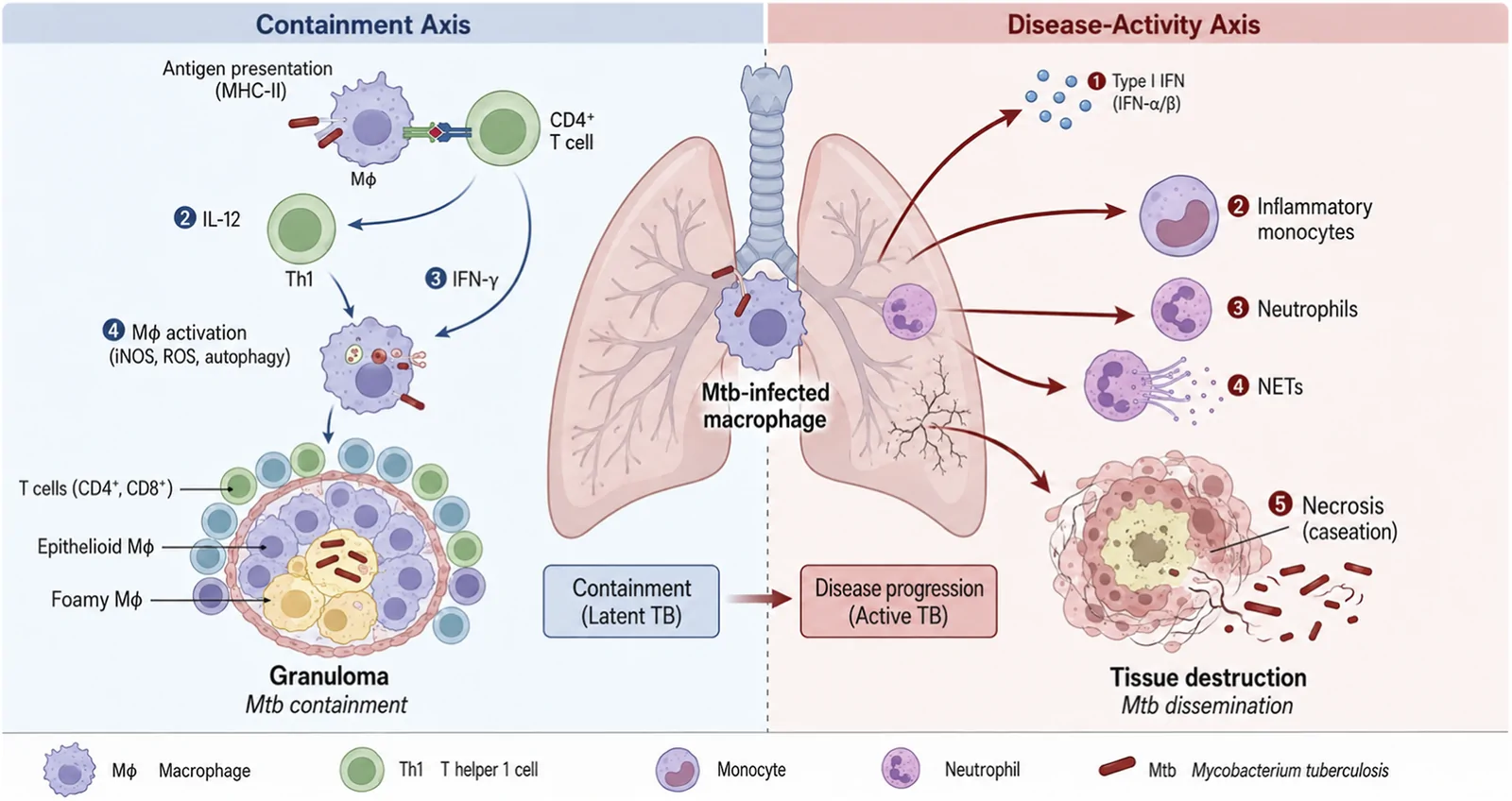
Immune-mechanistic architecture of host-response biomarkers in pulmonary tuberculosis.

Type I interferon biology is central to this signal. Experimental studies show that type I interferon can promote early TB susceptibility, neutrophil recruitment, neutrophil extracellular trap release, plasmacytoid dendritic cell activation, and impaired lung T-cell-macrophage interactions (Kotov et al., 2023; Chowdhury et al., 2024; Lee et al., 2024; Branchett et al., 2025). Granuloma-focused single-cell profiling further shows that tissue lesions contain heterogeneous macrophage, neutrophil, dendritic-cell and lymphoid states, reinforcing the idea that blood RNA signatures reflect systemic correlates of complex tissue immunopathology rather than a simple measure of protective immunity (Seto et al., 2025). Using pulmonary tuberculosis as a representative model, these opposing immune programs provide the mechanistic basis for interpreting host-response transcriptomic biomarkers as indicators of immune containment or disease activity, as illustrated in Figure 1. These containment and disease-activity programs can coexist; therefore, a stronger blood inflammatory signal should not be interpreted as evidence of more effective protective immunity.

Using pulmonary tuberculosis as a representative model, this schematic shows how protective containment and disease-activity programs shape host-response biomarker signals. The containment axis involves macrophage antigen presentation, IL-12/Th1 activation, IFN-γ-mediated antimicrobial function and organized granuloma formation. The disease-activity axis involves type I interferon signaling, inflammatory monocyte expansion, neutrophil recruitment, NET release, necrosis and tissue destruction. Abbreviations: Mtb, *Mycobacterium tuberculosis*; Mφ, macrophage; Th1, type 1 helper T cell; IFN, interferon; NET, neutrophil extracellular trap.

## 3 Clinical translation across the TB continuum

A first translational use case is sputum-independent triage or diagnosis of symptomatic TB. Prospective multicenter head-to-head validation studies have compared multiple published host blood transcriptomic signatures using real-time PCR and found that several signatures can distinguish pulmonary TB from other respiratory diseases, although diagnostic accuracy varies across cohorts and comparator groups (Mendelsohn et al., 2022). In African symptomatic cohorts, blood RNA signatures showed clinical promise but did not uniformly meet ideal target-product-profile thresholds, indicating that they are best viewed as triage or pathway-enrichment tools rather than replacements for microbiological confirmation (Muwanga et al., 2024). Comparator design is crucial: the multicenter PCR and African multicohort studies included symptomatic other respiratory disease (ORD) controls, whereas some discovery datasets relied mainly on healthy or latent-infection controls, which can overestimate real-world specificity (Mendelsohn et al., 2022; Muwanga et al., 2024).

People living with HIV and patients with extrapulmonary TB represent priority populations. Blood RNA biomarkers in people living with HIV before antiretroviral therapy initiation did not outperform C-reactive protein sufficiently to be considered stand-alone triage tests in that setting, highlighting the specificity problems caused by HIV-associated immune activation and intercurrent infections (Mann et al., 2024). In TB lymphadenitis and pericarditis, blood RNA signatures outperformed C-reactive protein but still did not meet optimal WHO target-product-profile criteria, again supporting a role as components of diagnostic cascades rather than isolated decision tools (Mann et al., 2025).

Additional host-response analytes may strengthen future panels. Cell-free RNA has been proposed as a blood-based host-response biomarker for TB, with a six-gene cfRNA model showing potential for TB detection across cohorts (Chang et al., 2024). Plasma proteomic studies have also identified tuberculosis-specific host protein signatures, supporting multi-analyte models that combine RNA, proteins and clinical covariates to improve specificity beyond transcriptomics alone (Schiff et al., 2024). Two recent machine-learning studies extended this approach: a five-gene-pair whole-blood model discriminated pulmonary TB from latent infection, pneumonia, lung cancer, pulmonary nodules and healthy controls, while a five-gene-pair cfRNA classifier achieved robust validation performance and retained high sensitivity in a small HIV-coinfected subgroup (Wu et al., 2026a; Wu et al., 2026b). These models support relative-expression gene pairs as a potentially batch-robust route to parsimonious assays, but both remain research-stage tools requiring independent prospective validation.

A second use case is prediction of progression from infection to active disease. Household-contact and incipient TB studies show that host-response signatures are most informative over short time horizons. In Southern Indian contacts, blood RNA and protein biosignatures helped distinguish subclinical and incipient TB states from household-contact controls (Sivakumaran et al., 2023). In Brazilian close contacts, several transcriptomic signatures predicted progression most strongly within approximately 6–9 months, while performance weakened over longer prediction windows (Mendelsohn et al., 2024). An individual participant data meta-analysis of single-gene transcripts also supports the concept that simple host transcripts can contribute to subclinical TB detection, although no single marker solves the heterogeneity of early disease (Greenan-Barrett et al., 2025).

Biomarker-guided prevention requires more than prognostic accuracy. The CORTIS trial showed that a transcriptomic risk signature could identify individuals at increased risk, but the clinical impact of risk-guided preventive therapy depends on treatment efficacy, program design and feasibility (Scriba et al., 2021). Longitudinal studies of RISK11 further show that positive transcriptomic risk states can be transient and may reflect intercurrent viral infection as well as TB progression biology (Mulenga et al., 2021). In people living with HIV, transcriptomic biomarkers can provide diagnostic and prognostic information, but background immune activation changes threshold behavior and implementation requirements (Mendelsohn et al., 2021). Mathematical modeling suggests that targeted preventive therapy may improve efficiency in some settings, but real-world utility depends on prevalence, assay cost, treatment uptake and health-system capacity (Sumner et al., 2021). Because transient viral infection may also elevate RISK11-like states, serial testing after approximately 2–4 weeks could be evaluated to confirm persistent activation before preventive therapy is initiated; this strategy still requires prospective validation.

A third use case is treatment monitoring and outcome risk assessment. During effective therapy, transcriptomic inflammation generally declines, and blood transcriptional trajectories can correlate with PET-based measures of lung inflammation (Odia et al., 2021). A 22-gene model has been proposed for predicting individualized treatment duration, and the TB27 model was developed to predict Mtb culture conversion, supporting biomarker-informed treatment adaptation as a future objective (Heyckendorf et al., 2021; Reimann et al., 2025). Non-transcriptomic host biomarkers, including CRP, IL-6, IP-10 and TNF-α, also decline during therapy and may provide complementary treatment-response information (Zimmer et al., 2022).

**FIGURE 2:**
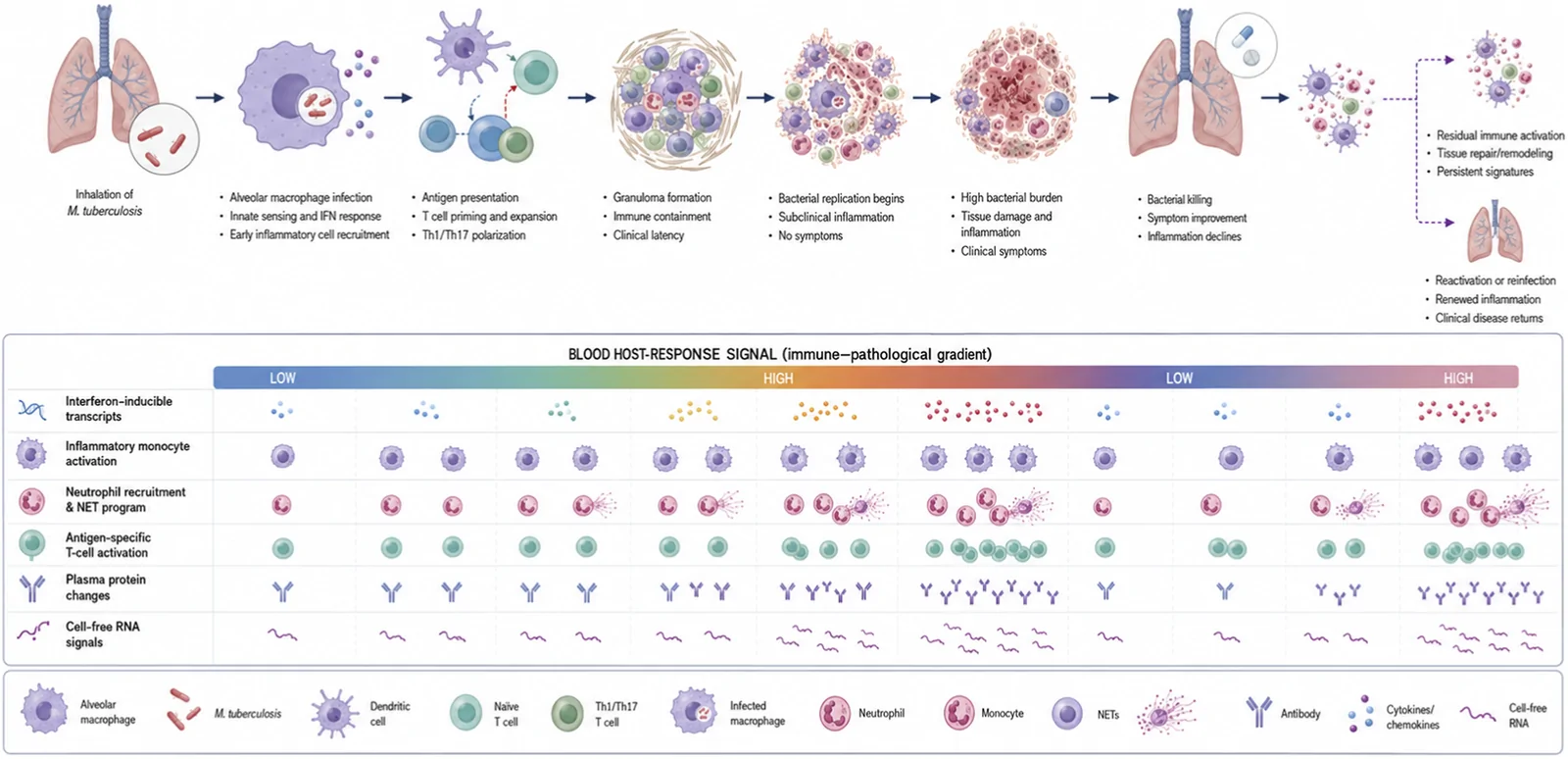
Dynamic host-response biomarker landscape across the pulmonary tuberculosis clinical continuum.

However, host-response signatures should not be treated as direct substitutes for microbiological cure. A recent analysis showed that resolution of TB blood RNA signatures failed to discriminate persistent sputum culture positivity after 8 weeks of treatment, and a linked commentary emphasized that blood RNA markers remain imperfect surrogates for 8-week sputum sterilization (Calderwood et al., 2024; Thu et al., 2024). For recurrence, the strongest current evidence suggests that host-response biomarkers may detect biologically active failure or recurrence better than they predict recurrence at baseline. In the Pan-African TB Sequel study, Mtb-specific T-cell activation markers and transcriptomic signatures identified treatment failure or recurrence after treatment completion, but end-of-treatment prediction remained limited (Bauer et al., 2026). In pulmonary TB, serial interpretation of host-response modules may support clinical risk stratification from early infection to active disease, treatment response and post-treatment recurrence surveillance, as summarized in Figure 2. Representative signatures and their decision contexts are compared in Table 1. Accordingly, host-response trajectories should trigger confirmatory microbiological, radiologic or clinical assessment rather than define cure or bacterial clearance on their own.

**TABLE 1:** Representative host-response transcriptomic signatures and translational use cases.

Signature/approach	Sample and platform	Intended clinical use	Evidence and strength	Main constraint/maturity
RISK6	Whole blood; 6-gene RT-qPCR	Triage/diagnosis, short-term progression and treatment monitoring	Multi-country and longitudinal validation (Penn-Nicholson et al., 2020; Bayaa et al., 2021)	Population-dependent thresholds and non-TB inflammatory overlap; intermediate maturity
RISK11/CORTIS	Whole blood; 11-gene RT-qPCR	Short-term progression enrichment and preventive-therapy targeting	Prospective validation and biomarker-guided randomized trial (Scriba et al., 2021; Mulenga et al., 2021)	Transient viral noise; benefit depends on treatment and program design; intermediate maturity
cfRNA 6-gene/5-gene-pair models	Plasma cfRNA; targeted expression or gene-pair models	Sputum-independent pulmonary TB diagnosis	Multicountry parent cohorts; AUC 0.947 for the gene-pair model (Chang et al., 2024; Wu et al., 2026b)	Specialized preanalytics and limited independent cfRNA validation; early maturity
PTBD 5-gene-pair model	Whole blood transcriptomes; rank-order machine learning	Triage/diagnosis and exploratory treatment-outcome prediction	Large heterogeneous dataset with ORD controls and external validation (Wu et al., 2026a)	Requires conversion to a locked targeted assay and prospective utility testing; early maturity
22-gene model/TB27	Whole-blood transcriptomic panels	Treatment-duration or culture-conversion assessment	Longitudinal treatment studies (Heyckendorf et al., 2021; Reimann et al., 2025)	Not a substitute for culture; limited interventional validation; early maturity
MAMS6, RISK6, Sweeney3 and CD38	Whole-blood RNA and antigen-specific T-cell activation	Treatment failure and post-treatment recurrence detection	Pan-African recurrence cohort (Bauer et al., 2026)	Small event numbers and limited end-of-treatment prediction; early maturity

Using pulmonary TB as the illustrative disease model, this schematic links infection, granuloma formation, subclinical inflammation, active disease, treatment response and recurrence to measurable blood host-response modules. Interferon-inducible transcripts, inflammatory monocyte activation, neutrophil/NET programs, antigen-specific T-cell activation, plasma protein changes and cell-free RNA signals increase with disease activity, partially normalize during effective treatment and may re-emerge during recurrence or reinfection. Abbreviations: Mtb, *Mycobacterium tuberculosis*; NET, neutrophil extracellular trap; cfRNA, cell-free RNA.

## 4 Toward clinical implementation in precision TB medicine

The translational value of host-response biomarkers depends on their linkage to specific clinical decisions. A triage biomarker should reduce unnecessary confirmatory testing while preserving sensitivity. A progression biomarker should enrich preventive therapy among those most likely to develop disease. A treatment-monitoring biomarker should identify patients who need adherence assessment, drug-resistance evaluation, imaging, extended therapy or intensified follow-up. These use cases require different thresholds, because high sensitivity is prioritized in triage, whereas higher specificity is needed for preventive therapy and recurrence surveillance. At present, evidence is most mature for triage and diagnostic pathway enrichment, moderate for short-term progression prediction and treatment monitoring, and early for recurrence surveillance.

Several implementation barriers remain. First, interferon-myeloid signatures are not TB-specific. Viral infections, bacterial pneumonia, HIV, autoimmune inflammation and recent vaccination may generate overlapping transcriptional programs. Second, disease and host heterogeneity affect performance. Diabetes comorbidity impairs resolution of treatment-associated blood transcriptomes, illustrating how metabolic disease can alter biomarker kinetics (Eckold et al., 2023). Systematic review data in people living with HIV similarly show that biomarker performance cannot be assumed to generalize from HIV-negative pulmonary TB cohorts (Mendelsohn et al., 2023). Third, RNA sequencing is useful for discovery but impractical for routine TB programs; simplified RT-qPCR, digital PCR or cartridge-compatible panels are required for clinical deployment. Children, extrapulmonary TB and geographically distinct populations likewise require subgroup-specific calibration and threshold validation rather than direct transfer of cutoffs developed in adult pulmonary TB cohorts.

Clinical translation also requires signature shrinkage from high-dimensional RNA-sequencing discovery data to stable, low-gene panels. Feature selection should be performed within training resamples using approaches such as penalized regression or ensemble-based variable-importance ranking, followed by stability assessment, locked thresholds and independent external validation. Relative-expression gene-pair models may reduce batch dependence, whereas conversion to RT-qPCR or cartridge formats still requires standardized normalization and analytical validation (Wu et al., 2026a; Wu et al., 2026b).

Recent integrative and review studies argue that the next stage should move from signature discovery to decision-based validation, assay standardization and implementation evaluation (Omrani et al., 2025; Cui et al., 2025). Cost-effective transcriptomic profiling may expand discovery and validation capacity, but it does not remove the need for clinical utility trials (Sun et al., 2024). Studies comparing Mtb DNA detection in peripheral blood mononuclear cells with incipient TB RNA signatures also show that host and pathogen biomarkers may capture different biological processes and should not be assumed to be interchangeable (Rosenheim et al., 2024).

## 5 Discussion and future directions

Host-response transcriptomic biomarkers have advanced substantially, but their near-term role is likely to be complementary rather than substitutive. They are most compelling as components of integrated precision pathways that combine symptoms, imaging, microbiology, clinical risk factors, comorbidities and host-response scores. For example, a high transcriptomic score in a symptomatic patient could prioritize molecular testing and radiography; a high short-term progression score in a household contact could trigger preventive therapy or intensified follow-up; and renewed host-response activation after treatment could prompt recurrence assessment.

Multi-omic integration is a major future direction. Protein biomarkers may improve operational feasibility because immunoassays are often easier to decentralize than RNA assays. Systematic review and meta-analysis of host blood protein biomarkers supports their value for screening, while plasma protein signatures have been associated with Mtb activity before and during treatment (Gaeddert et al., 2024; Ndiaye et al., 2022). Immune protein panels have also been shown to predict progression among household contacts, and recent proteomic studies identified plasma protein biomarkers linked to progression or recent infection (Rajamanickam et al., 2025; Han et al., 2026a; Han et al., 2026b). These findings support a future in which transcriptomic, proteomic and antigen-specific T-cell activation markers are combined in lean, clinically calibrated panels. These modalities occupy different stages of translation: RNA sequencing and broad proteomics remain primarily discovery and validation tools, whereas small RT-qPCR or immunoassay panels are more deployable. A single-cartridge RNA-protein platform would also face incompatible sample-preparation chemistries, calibration and quality-control requirements, cold-chain and reader constraints, and higher per-test costs; staged or reflex testing may therefore be more realistic for low- and middle-income countries in the near term.

The field should now prioritize five tasks: prospective multicenter validation in high-burden settings; subgroup-specific calibration for HIV, diabetes, children and extrapulmonary TB; standardized pre-analytical workflows; platform simplification; and interventional studies showing that biomarker-guided pathways improve outcomes. Recent reviews emphasize the same transition from biomarker discovery to actionable diagnostic, monitoring and immunity frameworks, including use of RISK6-like signatures for diagnosis, treatment monitoring and risk stratification (Barnie et al., 2025; Mamudi et al., 2026).

## 6 Conclusion

Host-response transcriptomic biomarkers provide a biologically coherent and clinically relevant approach to precision TB. Their core signal reflects an interferon-myeloid-neutrophil inflammatory axis that emerges during progressive and active disease, attenuates during effective treatment, and may reappear during failure or recurrence. The strongest translational opportunities are sputum-independent triage, short-term progression risk stratification, treatment-response assessment and post-treatment surveillance. Nevertheless, clinical implementation requires specificity against non-TB inflammation, validation in heterogeneous populations, simplified assays and evidence that biomarker-guided decisions improve patient outcomes. Precision TB medicine will therefore depend not on transcriptomic signatures alone, but on integrated pathways linking host biology, pathogen detection and clinical decision-making.
